# Enhanced Visualization of Fine Needles Under Sonographic Guidance Using a MEMS Actuator

**DOI:** 10.3390/s150203107

**Published:** 2015-01-30

**Authors:** Zhiyuan Shen, Yufeng Zhou, Jianmin Miao, Kien Fong Vu

**Affiliations:** 1 School of Mechanical and Aerospace Engineering, Nanyang Technological University, 50 Nanyang Ave., Singapore 639798, Singapore; E-Mails: shenyuan675603@gmail.com (Z.S.); jmiao@pmail.ntu.edu.sg (J.M.); 2 Department of Gastroenterology, Tan Tock Seng Hospital, 11 Jalan Tan Tock Seng, Singapore 308433, Singapore; E-Mail: charles_vu@ttsh.com.sg

**Keywords:** biopsy, ultrasound imaging, acoustic interference, MEMS actuator

## Abstract

Localization of a needle tip is important for biopsy examinations in clinics. However, the needle tip is sometimes difficult to discern under the guidance of sonography due to its poor visibility. A mini actuator that radiates a low-intensity ultrasound wave was manufactured using micro-electro-mechanical system (MEMS) technology. Interference between the radiated and diagnostic ultrasound pulses was observed as bright lines in the B-mode ultrasound image, from which the mini actuator could be recognized with ease. Because the distance between the mini actuator and the needle tip is fixed, the needle tip can be determined despite its inconsistent appearance in the sonography. Both gel phantom and *ex vivo* tissue evaluation showed that the needle tip can be determined reliably utilizing the acoustic interference pattern.

## 1. Introduction

Fine needle biopsy or aspiration is widely used during diagnosis, either extracorporeally via the skin or intracorporeally (*i.e.*, in conjunction with various endoscopes). This technique is safer and less traumatic than an open surgical biopsy [1,2]. In addition, fine needle intervention can also be used in therapy (*i.e.*, needle-based cancer ablation by microwave and radiofrequency), which is poised for substantial and sustainable growth [3,4]. The operator is able to view the biopsy needle or wire in real time as it advances to the target using existing imaging modalities (*i.e.*, CT, MRI, and ultrasound). Among these imaging modalities, ultrasound is preferred due to its technical advantages, such as non-ionization, low cost, mobility, and high frame rate [5,6]. However, needle localization under the guidance of ultrasound imaging is sometimes difficult, due to its poor visibility. During image-guided renal biopsy the accuracy of CT was 93.8%, while the corresponding value for sonography was 76.4% [7]. The frequency of adequate specimen cellularity of thyroid biopsy under the guidance of sonography varied from 68% to 96.6% [8]. Biopsy misplacement may result in multiple insertions, lengthy procedures, injuries to other tissues or organs, and repeated procedures for incomplete interventions. Misconduct in biopsy interventions are estimated to cost over $1 billion in healthcare expenditures annually [9].

Several approaches have been developed to improve the visualization of fine needles under the guidance of sonography. Two-dimensional real-time freehand ultrasound imaging can be tagged with position data to obtain a three-dimensional volume of the target and the surrounding structure for navigation [10]. Meanwhile, multiple sensors (*i.e.*, electromagnetic and optical sensors), including the sensor embedded in the ultrasound probe and the catheter, are used to keep track of the distal tip of the needle in a positioning system (*i.e.*, a magnetic field based system) during the procedure and display the current position and the predicted trajectory in real time [11]. The position accuracy was estimated to be 0.71 ± 0.43 mm in a non-surgical environment with a maximum error of 2.96 mm [12]. It is also found that the deflection from the intended path increases with the penetration depth due to an angular incidence of the biopsy needle [13]. However, all of these approaches require complicate hardware which may prevent their wide application. Therefore, an easy and reliable method of localizing the biopsy needle is in great need.

In this study, a new method for enhancing the visualization of the biopsy needle in the sonography is proposed and tested. A mini ultrasonic actuator was manufactured using micro-electro-mechanical system (MEMS) technology and attached to the needle tip. Its resonance spectrum was measured and compared with the simulated displacement using the finite element method. The acoustic pressure was also measured using a calibrated hydrophone. Once activated, the actuator produces bright interference in the sonography in both gel phantom and *ex vivo* tissues. From this interference, the location of the mini actuator and the needle tip can be accurately determined in a convenient and reliable manner.

## 2. Methodology

### 2.1. Fabrication

**Figure 1.**
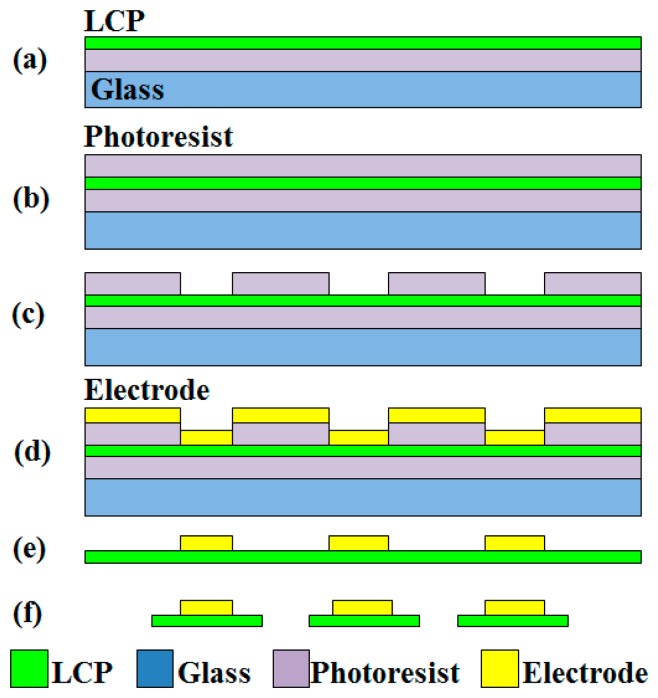
Flow chart depicting the fabrication process of the flexible electrode: (**a**) Liquid crystal polymer (LCP) bonded with glass wafer; (**b**) spin-coating of the photoresist; (**c**) patterning of the photoresist; (**d**) sputtering of the electrode; (**e**) patterning of the electrode, and (**f**) cutting of the electrode into strips.

The mini ultrasonic actuator was made of a piezoelectric ceramic element embedded between two flexible electrodes. A piezoelectric ceramic sheet (PSI-5A4E, Piezo Systems Inc., Woburn, MA, USA) with a thickness of 127 μm was sputtered with 300 nm Au and 30 nm Cr as electrodes on both sides; the chromium layer served as a buffer layer. Afterwards, the sheet was cut into 1 mm × 1 mm dies using a dicing saw (DAD552, Disco, Tokyo, Japan). The element would vibrate in the transverse length mode and excite ultrasonic waves. A liquid crystal polymer (LCP; ULTRALAM 3850, Rogers Corp., Rogers, CT, USA) with a thickness of 100 μm was adopted as a substrate to fabricate flexible electrodes. The electrodes had a width of 70 μm and a length of 6 cm, with a 1 mm × 1 mm pad at one end. A lift-off process was used to pattern the electrodes, as shown in Figure 1. The LCP was bonded with a 4-inch glass wafer using photoresist. Another 4.5 μm photoresist was spin-coated on top of the LCP. The photoresist was pre-baked for 4.5 min, exposed for 18 s in a lithography machine (MA6, Karl Suss, Garching, Germany), and developed for 2 min. Finally, layers of 10 nm Cr and 300 nm Au were then sputtered on the wafer bearing the cured patterned photoresist.

**Figure 2.**
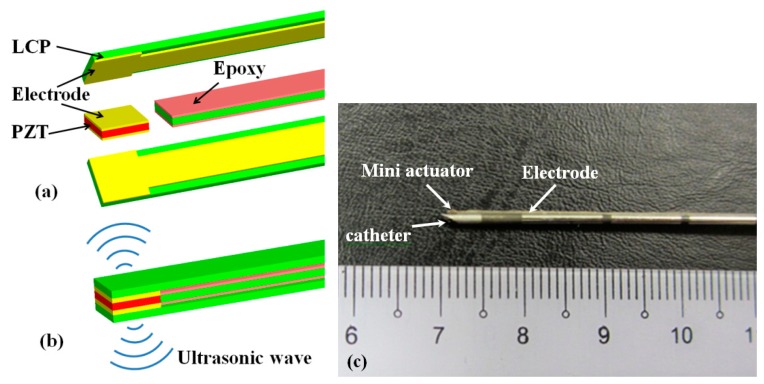
Sketches of: (**a**) the lead zirconate titanate (PZT) element being bonded between two flexible electrodes patterned on LCPs and (**b**) the assembled device; and (**c**) a photo of the ultrasound mini actuator attached to an 18 G biopsy needle.

The LCP with patterned electrodes was cut into 1 mm × 6 cm wide strips. Two LCP strips were bonded face-to-face using an epoxy with another bare LCP strip embedded between those strips as an insulation layer (as shown in Figure 2). The lead zirconate titanate (PZT) die was bonded in between the two electrode pads using a conductive epoxy as the intermediate layer. After bonding, the whole device was coated with a thin layer of polydimethylsiloxane (PDMS) precursor (Sylgard 184, Dow Corning, Midland, MI, USA) and then cured in an oven at 80 °C for 2 h. The PDMS thin film is biocompatible and will isolate the device from conductive body fluids. After assembly, the PZT was poled under a 225 V voltage for 5 min and then a 450 V voltage for 15 min to achieve a piezoelectric effect. The flexible electrode was conformably attached to an 18 G biopsy needle (US Biopsy, Franklin, IN, USA) using epoxy with the piezoelectric actuator that locates at the needle tip (see Figure 2c). Both the materials and the processes used in the manufacture are well established.

### 2.2. Characterization

The impedance spectra of the PZT actuator were measured by an impedance analyzer (4294A, Agilent Technologies, Santa Clara, CA, USA) with a frequency range of 0.1 MHz to 10 MHz. The performance of the PZT actuator was simulated using the acoustic module of a finite element method software (COMSOL 4.2a, Comsol, Burlington, MA, USA). The average displacement across the surface, which was obtained by sweeping the frequency from 1 MHz to 10 MHz, was used to determine the resonant characteristics and make comparison with the measurement results. The PZT element was driven by a function generator (AF3021B, Tektronics, Beaverton, OR, USA) at the amplitude of 10 V and the measured resonant frequency. The emitted acoustic pressure was detected using a calibrated miniature broadband hydrophone (MHA9, Force Technology, Brøndby, Denmark) placed at a distance of approximately 1 mm from the ultrasonic actuator which was immersed in an acoustic testing tank filled with degassed and deionized water (oxygen concentration < 4 mg/L, T = 25 °C, as measured by DO700, Extech Instrument, Waltham, MA, USA). The maximum pressure was determined by manually scanning the hydrophone which was attached to a three-axis translation stage.

### 2.3. Visualization

Visualization of the biopsy needle was evaluated using an ultrasound imaging system (SonixTouch, Ultrasonix, Vancouver, BC, Canada) with a convex array transducer (C7-3/50, Ultrasonix). The inital evaluation was carried out using a breast phantom (BP1901, Blue Phantom, Redmond, WA, USA) which contains a broad range of elastic masses and has acoustic and physical properties that are similar to those of real tissue. Furthermore, fresh porcine kidney purchased from a local slaughterhouse (Primary Industries Pte Ltd, Singapore, Singapore) was embedded in an agarose-starch mixed gel and used in the *ex vivo* investigation. The distances between the assumed biopsy tip (*i.e.*, the end of the hyperechoic pixels) and the actuator (*i.e.*, the starting site of interference) in the sonography were measured in both gel phantom and porcine kidneys using the built-in caliper capability. The obtained distances were then compared with the actual results using a digital caliper (digiMax, Wiha, Werkzeuge GmbH, Schonach, Germany) on the needle. The sample size for each group was five.

## 3. Results

### 3.1. Resonance and Radiated Pressure

**Figure 3.**
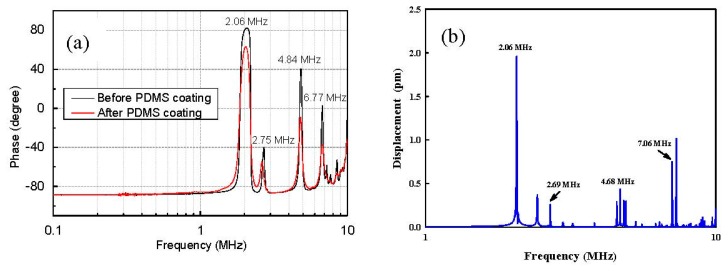
(**a**) Impedance spectrum of the PZT element in the frequency domain up to 10 MHz; and (**b**) the simulated average displacement across the surface of the PZT element using the finite element method.

The impedance of the PZT actuator is shown in Figure 3a. The PDMS thin film had a minor damping effect on the transducer, as illustrated by the slightly reduced *Q* factors of the resonances (*i.e.*, only 2.2% at 2.06 MHz). Meanwhile, the fundamental resonant frequency was shifted slightly from 2.08 MHz to 2.06 MHz. However, attaching the actuator to the biopsy did not change the electrical impedance significantly (difference < 2%). Similarities were found between the simulation and the measurement for the resonances (*i.e.*, the fundamental resonance and other high frequency modes in Figure 3). Because of the low attenuation of the materials and the absence of a bonding layer in the model, the simulated resonant peaks are much sharper. The discrepancies that were observed between the simulation and measurement for the higher frequency modes (*i.e.*, 4.68 and 7.06 MHz *vs.* 4.84 and 6.77 MHz, respectively) may be due to the uncertainty of the epoxy thickness between the flexible electrode and the needle, which cannot be determined experimentally. The maximum acoustic pressure was near the center of the actuator and was measured to be 12.3 ± 1.5 kPa in the free field at a driving voltage of 10 V and a resonant frequency of 2.06 MHz.

### 3.2. Gel Phantom and Ex Vivo Evaluation

**Figure 4.**
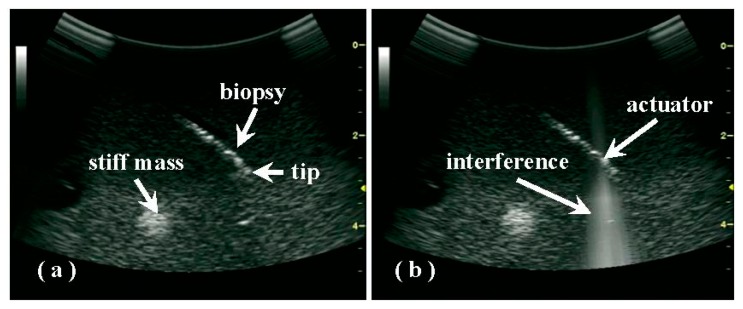
A comparison of sonography-guided biopsy in a breast gel phantom with both hyper- and hypo-echoic lesions (**a**) before and (**b**) after the activation of the mini actuator, where the interference generates bright lines.

Although the acoustic pressure produced by the actuator was quite weak, the interference that occurred with the diagnostic pulses could also be detected by the convex array and diagnostic circuit. When the actuator was turned on, bright and clear lines in a conical shape appeared in the sonography; the location of the actuator could be easily determined from these lines (see Figure 4). After changing the ultrasonic emission from continuous to pulsed mode, the interference appeared to be flashing (data not shown).

Because of speckle noise in the sonography, the biopsy needle did not appear as continuous hyper-echoic pixels, but rather appeared as discrete ones (Figure 4). Therefore, the needle tip, which may be below the sonographic resolution, was not determined easily and reliably using conventional B-mode imaging, especially inside a target with many hyperechoic scatters and significant heterogeneities. Due to its small size, the actuator itself does not show up in the sonography and introduce any shadowing effect. It is found that with increasing sample heterogeneity and structural complexity, the variation in the distance between the PZT actuator and the assumed needle tip, as measured using the built-in caliper in the sonography increased from 2.9 ± 0.6 mm in the gel phantom to 2.6 ± 1.4 mm in *ex vivo* tissue (as listed in Table 1). Thus, extending from the site of interference by the predetermined distance (*i.e.*, the actual value between the actuator and the needle tip) along the biopsy orientation may be an easy method of estimating the position of the biopsy tip, which may not always be visible in the sonography.

## 4. Discussion

Due to the popularity of biopsies in the clinic, guidance during these interventions is critical for the safety and efficacy of these procedures. Sonography guidance is preferred to the fluoroscopic approach primarily due to the low cost and the absence of ionization for the sonographer. However, speckle noise in sonography and low contrast limit the visualization ability. A sharp needle makes the puncture easy, but the small tip of such a needle may be below the resolution of sonography. Although a high ultrasound frequency improves the resolution, the penetration depth will be limited. In this study, a mini ultrasonic actuator manufactured using MEMS technology was attached to the needle tip. Due to its small size, the actuator itself does not introduce any artefacts in the sonography. An ultrasound burst was radiated, and its spatial-peak temporal-average acoustic intensity (5 mW/cm^2^) is far below the safety threshold defined by the regulation of the United States Food and Drug Administration (FDA). Bright interference lines could be clearly identified in the sonograph, from which the location of the actuator could be determined easily and reliably. As a result, the accuracy of sampling tissues from the region of interest could be increased without damaging the intervening vital parts (*i.e.*, vessels, nerves, and the fetus), and the number of biopsy insertions could be reduced. In comparison to current approaches, our proposed method can be applied to all commercially available ultrasound imaging systems without software implementation. Closeness of ultrasound imaging system is a great barrier to the development and application of new programs, such as biopsy navigation using a magnetic positioning system [11,14].

MEMS technology promotes the development and mass manufacture of small sensors with high reliability and consistent performance at a low cost for disposable use [15]. More specifically, integrated electronics may simplify board-level system design for additional functionalities. For example, in principle, the mini ultrasonic element can also work as a receiver to form an A**-**mode line for detecting the distance between biopsy and vital tissue with an on-chip transmit-and-receive circuit.

Interference is a common phenomenon in acoustics. In high-intensity focused ultrasound (HIFU) therapy, interference in the sonography will disable lesion monitoring and must be avoided [16]. Thus, HIFU is usually delivered in burst mode, and the detection of the lesion and its boundaries should be performed only during the interval. Similarly, hyperechoic interference affects the diagnosis of tissues and structures in the radiation region of the actuator. Changing the driving signal from continuous mode to pulsed mode allows the interference to appear in a flashing manner with a pattern that is determined by the pulse duration and the pulse repetition frequency. However, the interference noise is also advantageous for localizing the acoustic source. It was used to determine the position of the HIFU focus, where the interference noise was maximally converged and enhanced [17]. When visualizing the biopsy needle in *ex vivo* tissue, the interference becomes weak due to the higher attenuation in the tissue than that in the phantom. Varying the amplitude and frequency of the driving signal and the time-gain compensation in the sonography would change interference brightness.

The design of actuator used in this study, including the materials and manufacture process, has not been optimized. If an acoustic matching layer is attached to the front surface of the piezoelectric material and an electrical impedance matching network, stronger radiation is expected. However, it is noted that even using such a weak acoustic output clear interference was observed in the sonography in both gel phantom and *ex vivo* tissue samples, confirming the validity of our concept. In addition, a large number of animal experiments are required to completely evaluate the accuracy and precision of this method and compare this method with the other existing approaches.

## 5. Conclusions

In summary, we have investigated a novel low-cost method for enhancing the visualization of fine biopsy needles under the guidance of ultrasound imaging by utilizing the acoustic interference phenomenon and MEMS technology. Although the fabrication of mini actuators is well established, integrating this technology with biopsies is conceptually new and provides an easy and reliable approach to enhance visualization in the sonography. Further evaluations will be carried out in the future in animals to determine for the potential of this approach for clinical translation. With slight modifications, this approach may be adopted for localizing catheters in the application of tumor ablations, angioplasty, anesthesia, nerve blocks, vascular access, and embryo transfer in the fertilizations, where alignment precision with respect to the target determine the outcome.

## Figures and Tables

**Table 1.** Distance between the PZT actuator and the tip of the biopsy needle.

**Caliper**	**Breast Phantom**	***Ex Vivo* Porcine Kidney**
2.8 ± 0.1 mm	2.9 ± 0.6 mm	2.6 ± 1.4 mm

## References

[1] Kim E.-K., Park C.S., Chung W.Y., Oh K.K., Kim D.I., Lee J.T., Yoo H.S. (2002). New sonographic criteria for recommending fine-needle aspiration biopsy of nonpalpable solid nodules of the thyroid. Am. J. Roentgenol..

[2] Seo M.S., Baker J.A., Rosen E.L. (2003). Sonographic detection and sonographically guided biopsy of breast microcalcifications. Am. J. Roentgenol..

[3] Rehman J., Landman J., Lee D., Venkatesh R., Bostwick D.G., Sundaram C., Clayman R.V. (2004). Needle-based alabtion of renal parenchyma using microwave, cryoablation, impedance- and temperature-based monopolar and bipolar radiofrequency, and liquid and gel chemoablation: Laboratory studies and review of the literature. J. Endourol..

[4] Thiagalingam A., Pouliopoulos J., Boyd A.C., Eipper V., Yung T., Ross D.L., Kovoor P. (2005). Cooled needle catheter ablation creates deeper and wider lesions than irrigated tip catheter ablation. J. Cardiovasc. Electrophsiol..

[5] Nyland T.G., Mattoon J.S., Herrgesell E.J., Wisner E.R., Nyland T.G., Mattoon J.S. (2002). Ultrasound-guided biopsy. Small Animal Diagnostic Ultrasound.

[6] Memel D.S., Dodd G.D., Esola C.C. (1996). Efficiency of sonography as a guidance technique for biopsy of abdominal, pelvic, and retroperitoneal lymph nodes. Am. J. Roentgenol..

[7] Ralls P.W., Barakos J.A., Kaptein E.M., Friedman P.E., Foulodian G., Boswell W.D., Halls J., Massry S.G. (1987). Renal biopsy-related hemorrhage: Frequency and comparison of CT and sonography. J. Comput. Assist. Tomogr..

[8] Rausch P., Nowels K., Jeffrey R.B. (2001). Ultrasonographically guided thyroid biopsy: A review with emphasis on technique. J. Ultrasound Med..

[9] (2008). European Market for Biopsy Devices.

[10] Sjølie E., Langø T., Ystgaard B., Tangen G.A., Nagelhus Hernes T.A., Mårvik R. (2003). 3D ultrasound-based navigation for radiofrequency thermal ablation in the treatment of liver malignancies. Surg. Endosc..

[11] McGrath J., Siegel D.N., Waldman D.L. (2013). Ultrasonography and GPS technology. Ultrasound Clin..

[12] Banovac F., Glossop N., Lindisch D., Tanaka D., Levy E., Cleary K. (2002). Liver tumor biopsy in a respiring phantom with the assistance of a novel electromagnetic navigation device. Medical Image Computing and Computer-Assisted Intervention—MICCAI 2002.

[13] Wooten W.J., Nye J.A., Schuster D.M., Nieh P.T., Master V.A., Votaw J.R., Fei B. (2013). Accuracy evaluation of a 3D ultrasound-guided biopsy system. Proc. SPIE.

[14] Hummel J., Figl M., Birkfellner W., Hfner M., Kollmann C., Bergmann H. Navigation system for flexible endoscopes.

[15] Keith J.R. (2004). Applications of MEMS in surgery. IEEE Proc..

[16] Owen N.R., Bailey M.R., Hossack J., Crum L.A. (2006). A method to synchronize high-intensity, focused ultrasound with an arbitrary ultrasound imager. IEEE Trans. Ultrason. Ferroelectr. Freq. Control.

[17] Wu C.-C., Chen C.-N., Ho M.-C., Chen W.-S., Lee P.-H. (2008). Using the acoustic interference pattern to locate the focus of a high-intensity focused ultrasound (HIFU) transducer. Ultrasound Med. Biol..

